# Comparative impacts of aboveground and belowground enemies on an invasive thistle

**DOI:** 10.1002/ece3.3751

**Published:** 2017-12-27

**Authors:** Krystal A. Nunes, Peter M. Kotanen

**Affiliations:** ^1^ Department of Ecology and Evolutionary Biology University of Toronto Mississauga Mississauga ON Canada

**Keywords:** aboveground interactions, *Cirsium arvense*, common garden, herbivory, invasive species, plant–herbivore interactions, plant–soil (belowground) interactions, soil mesofauna

## Abstract

Most research examining how herbivores and pathogens affect performance of invasive plants focuses on aboveground interactions. Although important, the role of belowground communities remains poorly understood, and the relative impact of aboveground and belowground interactions is still debated. As well, most studies of belowground interactions have been carried out in controlled environments, so little is known about the role of these interactions under natural conditions or how these relationships may change across a plant's range. Using the invasive plant *Cirsium arvense*, we performed a reciprocal transplant experiment to test the relative impacts of above‐ and belowground interactions at three sites across a 509‐km latitudinal gradient in its invaded range in Ontario, Canada. At each site, *C. arvense* seedlings were protected with above‐ and/or belowground exclosures in a factorial design. Plant performance (biomass, height, stem thickness, number of leaves, length of longest leaf, maximum rhizome length) was greatest when both above‐ and belowground exclosures were applied and lowest when no exclosures were applied. When only one type of exclosure was applied, biomass generally improved more with belowground exclosures than with aboveground exclosures. Despite site‐to‐site differences in foliar damage, root damage, and mesofaunal populations, belowground interactions generally had a greater negative impact on performance than aboveground herbivory alone. These results stress the importance of including both aboveground enemy interactions and plant–soil interactions in studies of plant community dynamics and invader performance.

## 1 INTRODUCTION

Interactions with natural enemies can potentially have important consequences for the success or failure of invasions by non‐native plants (Elton, 1958; Keane & Crawley, 2002; Torchin & Mitchell, 2004). However, most research investigating the effect of herbivores and pathogens on plant performance focuses on aboveground interactions, despite a growing consensus that belowground interactions may be very important (Dawson, Schrama, & Austin, 2016; van der Putten, Vet, Harvey, & Wackers, 2001), often having a greater impact on plant performance than aboveground damage (Barber, Adler, & Bernardo, 2011). It has been well established that plants heavily attacked by aboveground enemies often show reduced growth and fecundity (e.g., Ang, Kok, Holtzman, & Wolf, 1995; Bacher & Schwab, 2000), but belowground antagonists may also impact plant fitness and survival directly by damaging root tissue (Barber et al., 2011; Maron, 1998; Strong et al., 1995) or indirectly by changing interactions with other organisms (Hol, Raaijmakers, Mons, Meyer, & van Dam, 2016; Soler et al., 2012; Wardle, 2002). For instance, root herbivory can reduce flowering and thus rates of pollinator visitation (Barber et al., 2011), and can alter attack by aboveground herbivores through induction of leaf chemical defenses (Bezemer, Wagenaar, van Dam, & Wackers, 2003). Belowground communities also harbor many plant mutualists, such as mycorrhizal fungi, and indirectly beneficial organisms such as detritivores and decomposers. As a result, net effects of the soil community may be positive, negative, or neutral (Ehrenfeld, Ravit, & Elgersma, 2005). Finally, as aboveground and belowground interactions may act independently or may interact to affect plant performance (Johnson, Mitchell, McNicol, Thompson, & Karley, 2013; Wardle, 2002), understanding their simultaneous effects on a potentially invasive host is a difficult task. A review by Wardle et al. (2004) found that depending on the system, aboveground herbivores can have variable effects on soil organisms through plant–mediated interactions; both positive and negative effects were possible outcomes. Aboveground herbivores may positively impact soil organisms by promoting compensatory growth in the host plant, or by adding nutrients to the soil via frass. Alternatively, negative impacts may occur if defenses are induced in belowground tissues, if host plant productivity is reduced due to low herbivore tolerance, or through selection for unpalatable plants over time. These differing results may be due to the high context dependency of patterns in aboveground–belowground linkages (Johnson et al., 2012).

Measurements of relative impacts of aboveground and belowground organisms can depend on the design of the study. In particular, whether a laboratory or field study is conducted can greatly affect results (Heinze, Sitte, Schindhelm, Wright, & Joshi, 2016; Johnson et al., 2012). However, most studies on aboveground–belowground interactions have been carried out in highly controlled environments (Bezemer, Graca, Rousseau, & van der Putten, 2004; Bezemer et al., 2003; Engelkes et al., 2008; Friedli & Bacher, 2001; Gange & Brown, 1989; Kostenko, van de Voorde, Mulder, van der Putten, & Martijn Bezemer, 2012; Masters & Brown, 1992; Moran & Whitman, 1990), and so little is known about these relationships under true field conditions. In one exception, Maron (1998) explored aboveground–belowground interactions in the field by suppressing herbivores on stands of *Lupinus arboreus* (bush lupine) for three growing seasons. Suppressing aboveground seed predators increased seed production, but positive effects on plant fitness of reduced belowground herbivores were not evident until the third year of the experiment. No statistical interaction was found between above‐ and belowground herbivores on plant fitness, although this experimental design did not account for the effects of above‐ or belowground pathogens, bacteria, or fungi.

Less still is known about how the relative importance of aboveground and belowground interactions changes across a plant's range. This is of particular interest for invasive plants undergoing range expansion, as the interactions between a host plant and its enemies can be an important determinant of future spread. The Enemy Release Hypothesis is a well‐established explanation for the success of many invasive plants, whereby the invader benefits from reduced enemy pressure in the invaded range as a consequence of leaving behind species‐specific herbivores and pathogens during the invasion process (Elton, 1958; Keane & Crawley, 2002; Torchin & Mitchell, 2004). However, the same principles may apply within the invaded range, as an exotic can experience reduced enemy pressure as it invades new locations and expands its latitudinal range. A decline in herbivory within marginal areas of the invaded range has been shown for some guilds of aboveground herbivores (Harvey, Nipperess, Britton, & Hughes, 2013; Kambo & Kotanen, 2014; Nunes, Cassin, & Kotanen, 2016) and has been shown cross‐continentally for belowground herbivores and pathogens (Yang et al., 2013). The way in which the above‐ and belowground biota vary across large spatial scales will have important consequences for the direction and rate of spread of an invader to novel locations (van der Putten et al., 2009).

In this study, we report results of an experiment investigating above‐ and belowground interactions *in situ* using naturally occurring enemy communities of an invasive thistle. This research tests (i) the relative importance of above‐ and belowground interactions on the performance of an invader; and (ii) how this relationship might change over large spatial scales. We particularly focus on interactions with potential herbivores (aboveground insects and soil mesofauna), although we acknowledge that other enemies, particularly microbes, may contribute to our results. We hypothesize belowground interactions will have a negative impact on plant performance equal to or greater than aboveground interactions. We also hypothesize that the aboveground–belowground relationship will change across the invaded range as above‐ and/or belowground enemies become less prevalent toward the northern range edge in response to unfavorable climatic conditions, scarce host populations, or a shorter colonization history (Lau & Suwa, 2016; van der Putten et al., 2009).

## 2 MATERIALS AND METHODS

### 2.1 Study organism

*Cirsium arvense* (L.) Scop. (Canada thistle) is a clonal perennial herb native to Eurasia that has been classified as a noxious weed in Canada and the United States (Ang et al., 1995; Moore, 1975). It is often found in disturbed areas with rich soils and high light availability, such as old fields, agricultural areas, and along roadways (Tiley, 2010). In 1959, a biological control program was implemented in Canada to reduce its spread (Schröder, 1980), although the success of this program has been variable at best. The majority of biocontrol efforts have focused on the use of aboveground herbivores (Cripps et al., 2011), while few belowground interactions have been explored as biocontrol measures for this invader. Locally common aboveground herbivores in Ontario include the leaf beetle *Cassida rubiginosa*, the seed‐eating weevil *Larinus planus* and fly *Terellia ruficauda*, the stem‐galling fly *Urophora cardui*, and the leaf‐ and stem‐sucking bugs *Philaenus spumarius* and *Poecilocapsus lineatus*; the most common belowground enemies in our study sites are unknown. *C. arvense* was selected as our focal species because it is a common, widespread, and aggressive invader, most of its aboveground enemies are known, and the control of this species is of economic importance.

### 2.2 Seed collection and germination

*Cirsium arvense* seeds collected from five clonal populations in each of the Newmarket, Haliburton, and Timmins regions of Ontario, Canada in late summer 2013 were stored frozen until needed in this experiment. Seeds were then surface sterilized and cold stratified for 5 weeks (4°C on moist filter paper placed inside Parafilm sealed petri dishes) before planting in double‐autoclaved Sunshine Mix #1 Professional Growing Mix (50 min cycle at 121°C maximum temperature). They were then transferred to a growth chamber for germination. Seeds, and later seedlings, remained in the growth chamber for 4 weeks at 25°C and a 16‐hr light cycle.

### 2.3 Soil collection and preparation

Double‐autoclaved potting soil was mixed at a ratio of 3:1 with double‐autoclaved sand to minimize potential effects of enhanced nutrient availability from the soil sterilization process (personal communication, H. Maherali), and to aid in the process of root removal (Callaway, Montesinos, Williams, & Maron, 2013). As a source for both soil inoculum and mesofaunal community sampling, live soil was collected from a depth of 30 cm at each of the three common garden locations. Subsamples were collected from 10 sites within the area of each garden and homogenized to obtain a thorough representation of the belowground community at that particular location, at the cost of loss of information about variation among subsamples (Reinhart & Rinella, 2016).

### 2.4 Common garden design

**Figure 1:**
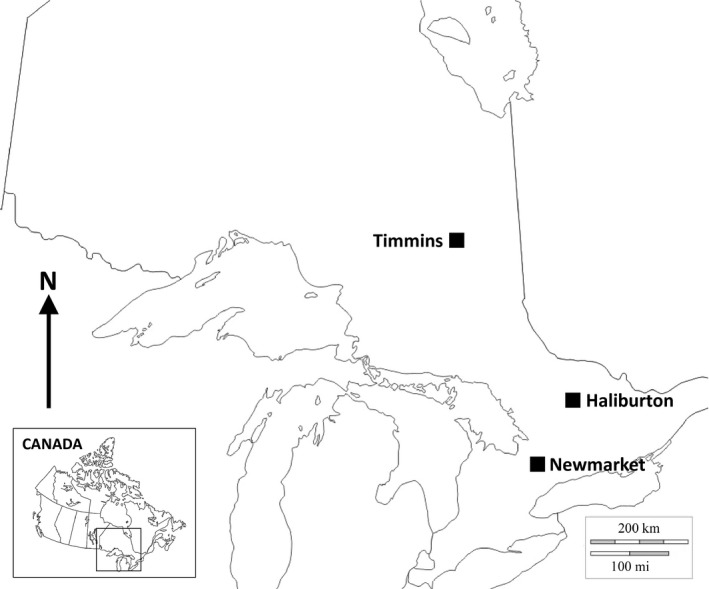
A map of Ontario, Canada indicating the three common garden locations across a 509‐km latitudinal distance

Replicate common gardens measuring 8 m by 14 m were created at three sites across a 509‐km latitudinal transect: Newmarket (44.027°N, 79.024°W), Haliburton (45.223°N, 78.593°W), and Timmins (48.388°N, 81.558°W; Figure 1). Gardens were installed on 27 May 2015, 30 May 2015, and 3 June 2015, respectively. Pots were randomized and placed into holes to accommodate the pot while leaving 5 cm above the soil surface; pots were placed 1 m apart.

*Cirsium arvense* seedlings were transplanted into round pots (21.6 cm tall by 21.6 cm wide) and randomly treated with above‐ and/or belowground exclosures in a fully factorial design. There were 21 replicates of each of the four treatment combinations for a total of 84 pots in each garden. A reciprocal transplant was incorporated into the experimental design with seven individuals in each exclosure treatment originating from one of the three regional seed sources. If we were to find that performance and/or damage differed more between garden sites than between plant provenances, this would suggest local environment is more important in determining herbivory levels than plant genotype (Garibaldi, Kitzberger, & Chaneton, 2011).

Half the pots were treated with a live soil inoculum collected from each of the common garden locations. Inoculum comprised 20% of the total soil volume; this ratio retains the mesofaunal component of the soil as well as the microbial species in a test of whole‐soil feedback, whereas a lesser ratio would generally capture microbial feedback only (Smith & Reynolds, 2015; Vandegehuchte, de la Pena, & Bonte, 2010). For the remaining pots, field soil was double‐autoclaved and incorporated into pots at the same ratio to create a belowground exclosure treatment. For the aboveground exclosure, three 122‐cm bamboo shoots were placed into each pot to support tubes of spun polyester. These tubes were made of AgroFabric PRO 17 grade fabric and allowed 95% light transmission. Pots without aboveground exclosures were also netted with tubes of spun polyester, but four vertical slits were made on each side of the tube 2.5 cm from the top of the exclosure to the soil surface; this allowed herbivore access to plants, but controlled for potential growth differences due to reduced sunlight transmission through the netting. Exposed areas of the soil surface were covered with 0.5 cm of autoclaved sand to reduce microbial colonization (per. comm., H. Maherali).

### 2.5 Plant sampling

Each garden was harvested 9 weeks after its respective installation date. Upon completion of the experiment, measures of plant growth (plant height, stem diameter at soil surface, number of leaves, and length of longest leaf) were quantified; plant survival rate was 100%. To quantify damage by aboveground herbivores, up to 10 leaves on each plant were randomly selected for visual estimates of herbivore damage. Although visual damage estimates can be highly accurate (Johnson, Bertrand, & Turcotte, 2016), sampled leaves were compared to digital leaves of known damage levels for calibration. Plants were also surveyed for the presence of galls caused by the stem galling fly *Urophora cardui*, although none were found. In the laboratory, roots were washed over a 1‐mm sieve, and the lengths of the taproot and longest rhizome were recorded. To quantify damage potentially caused by belowground herbivores, the number of root lesions per cm of taproot was recorded for each plant; root lesions were identified by areas of physical damage and/or decayed tissue. Above‐ and belowground biomass were harvested separately, dried at 60°C for 3 days and weighed.

### 2.6 Mesofaunal extraction

Quantifying microbial populations was beyond the scope of this experiment, but we were able to sample the mesofaunal component of the belowground environment; consistent with our aboveground and root sampling, this approach focuses on the faunal component of the thistle‐associated community. The belowground mesofaunal community (phyla Arthropoda, Annelida, and Nematoda) was surveyed immediately following the completion of the experiment by placing 700 ml of soil from each pot in a Berlese‐Tullgren extractor for 4 days. Specimens were preserved in 95% ethanol and identified to morphospecies.

### 2.7 Statistical analyses

Plant traits (above‐ and belowground biomass, plant height, rhizome length, etc.) and above‐ and belowground herbivory rates were analyzed using a three‐way ANOVA with exclosure treatment, garden location, and plant origin as fixed factors. We treated all four exclosure treatments as levels of a single factor rather than two crossed factors (above‐ × belowground exclusion) both to simplify the design and because we were interested in comparing four treatments against each other, rather than investigating interactions between them. Biomass was log‐transformed to meet the assumption of normality of residuals and to correct for unequal variances. Aboveground biomass did not meet the assumption of variance homogeneity (F_35,215_ = 1.57, *p* = .030; results reported from Brown‐Forsythe test for equality of group variances). Therefore, results for aboveground biomass should be treated with caution. Means were compared using Tukey's HSD tests.

To determine the relative importance of plant genotype versus local environment on rates of herbivory, an ANOVA was used to test the effect of plant origin, garden location, exclosure treatment, and their interactions on mean percent area leaf damage and mean number of root lesions per cm of taproot.

To identify differences in the belowground community between gardens, ANOVA and Tukey's HSD tests were performed on mesofaunal abundance and diversity for the live soil treatment. To describe differences in community composition, a nonmetric multidimensional scaling (NMDS) ordination plot was created using Bray–Curtis percent dissimilarity of standardized abundances. The amount of overlap in 95% confidence interval ellipses between common garden locations reflects community composition similarity. For each garden location, species accumulation curves were produced to determine the efficacy of the mesofaunal community sampling.

All analyses were performed in R (R Development Core Team 2016, version 3.3.0). Means are reported as ± standard error.

## 3 Results

### 3.1 Effect of treatments on plant performance

**Figure 2:**
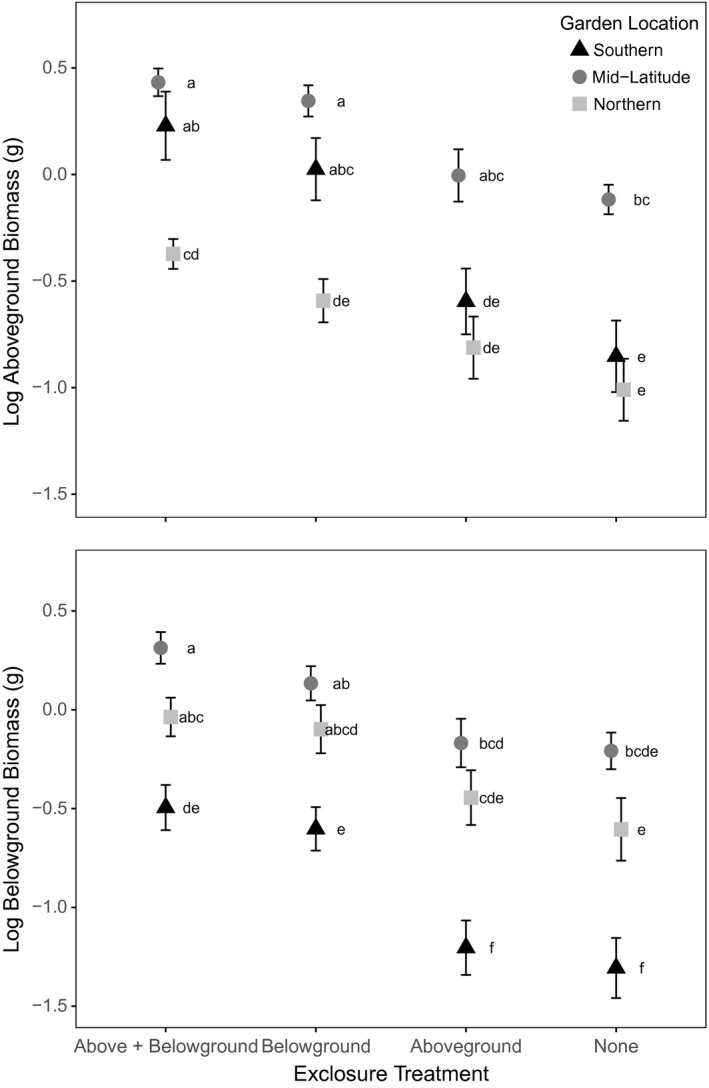
Aboveground (upper graph) and belowground (lower graph) mean ± SEM biomass of experimental *C. arvense* following a 9‐week growing period. Data points that do not share the same letter are significantly different (Tukey's grouping, *p *<* *.05)

The duration of our experiment was sufficient to detect numerous differences in growth among experimental treatments (Figure 2). The exclosure treatments had a significant effect on aboveground (F_3,215_ = 38.91, *p* < .0001), belowground (F_3,216_ = 34.95, *p* < .0001), and total plant biomass (F_3,215_ = 41.90, *p* < .0001; not shown). Despite a three‐way interaction for aboveground biomass only (F_12,215_ = 2.77, *p* = .002) and several two‐way interactions (Table S1), there were still clear and significant overall differences among treatments as indicted by Tukey's tests. At all locations, biomass was greatest when both aboveground and belowground exclosures were applied, and lowest when no exclosures were applied; when either an aboveground or belowground exclosure was applied, an intermediate level of plant growth was observed (Figure 2). However, the improvement in plant performance generally was greater when belowground exclosure treatments were applied than for those netted aboveground but still exposed to live soil. In four of six comparisons (three root biomass and three shoot biomass), belowground exclusion significantly increased growth relative to unprotected plants, but this was never true when adding aboveground protection alone (Tukey's HSD, *p* < .05; Figure 2).

*Cirsium arvense* plants in the mid‐transect site (Haliburton) typically grew to have the greatest biomass. Although consistently smaller than Haliburton plants, those grown in the southern garden (Newmarket) invested more in aboveground growth than those grown at the northern site (Timmins), which invested more in belowground growth (Tukey's HSD, *p* < .05; Figure 2).

The exclosure treatments also significantly affected plant height, although this effect was highly dependent on garden location (F_6,216_ = 3.30, *p* = .004; Figure S1). Plants in the most northern site grew to be the shortest (4.5 ± 0.2 cm) and showed no significant differences among treatments (Tukey's HSD, *p* > .05). Plants grown in the mid‐latitude and most southern site grew to be much taller (13.2 ± 0.6 cm and 10.7 ± 0.6 cm, respectively). There were significant treatment effects at these sites; plants that were treated with both above‐ and belowground exclosures grew to be the tallest (11.8 ± 0.8 cm), while those exposed to both above‐ and belowground interactions were the shortest (7.4 ± 0.5 cm). Plants treated with either a belowground (10.0 ± 0.8 cm) or aboveground (8.8 ± 0.6 cm) exclosure grew to an intermediate height. The same patterns were found in other measures of growth, as the effect of exclosure treatment had a significant effect on the number of leaves (F_3,216_ = 18.0, *p* < .0001), length of longest leaf (F_3,216_ = 18.3, *p* < .0001), and stem diameter (F_3,216_ = 18.5, *p* < .0001).

There was no significant treatment effect on taproot length (F_3,216_ = 1.59, *p* = .192), although the length of the longest rhizome significantly differed between exclosure treatments (F_3,216_ = 4.97, *p* = .002) and garden locations (F_2,216_ = 28.72, *p* < .0001) with no significant interactions. As with other measures of performance, rhizome length was greatest when both above‐ and belowground interactions were excluded, and shortest when no exclosures were applied. When either an above‐ or belowground exclosure was applied, intermediate rhizome length was observed. Plants grown in the mid‐latitude garden had significantly longer maximum rhizome length (36.3 ± 1.9 cm) than either the northern (24.3 ± 1.4 cm) or southern (23.1 ± 1.1 cm) garden locations.

### 3.2 Effect of reciprocal transplant

**Figure 3:**
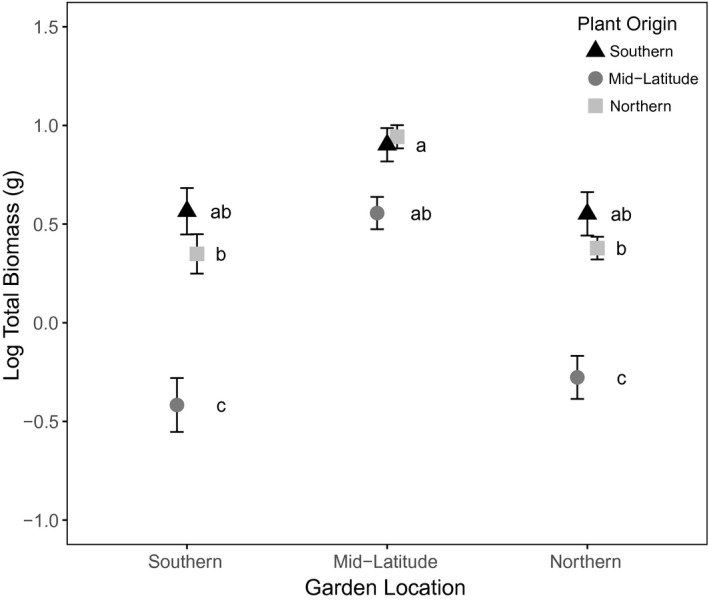
Result of reciprocal transplant on *C. arvense* total biomass following a 9‐week growing period. Data points show mean ± SEM biomass for plants of each seed origin separated by garden location. Data points that do not share the same letter are significantly different (Tukey's grouping, *p *<* *.05)

Plant performance did differ between common gardens and seed sources, but there was little evidence of local adaptation. Despite the small sample size (*n* = 7 for each plant origin per treatment, per garden), the effect of plant origin had a significant effect on total plant biomass (F_2,215_ = 73.49, *p* < .0001), although this was dependent on garden location and treatment (garden location × treatment × plant origin interaction, F_12,215_ = 2.27, *p* = .01; Figure 3). In all three gardens, seeds sourced from the mid‐latitude region resulted in plants that performed most poorly, while seeds sourced from the more southern and northern regions showed better performance. Although the mid‐latitude seedlings performed best in the mid‐latitude common garden, this was not evidence of local adaptation as all three seedling types showed the greatest biomass at this location (Figure 3).

Plant origin also had a significant impact on plant height. Seedlings sourced from the southern region grew the tallest (11.8 ± 0.7 cm) regardless of growing location or exclosure treatment (F_2,216_ = 29.64, *p* < .0001). Seeds sourced from the northern region produced plants of intermediate height (9.4 ± 0.6 cm), while seeds sourced from the mid‐latitude region produced the shortest plants (7.3 ± 0.5 cm). The effect of plant origin held true for the number of leaves (F_2,216_ = 8.3, *p* = .0003), length of longest leaf (F_2,216_ = 42.7, *p* < .0001), and stem thickness (F_2,216_ = 19.7, *p* < .0001).

There was no effect of plant origin on tap root length (F_2,216_ = 2.86, *p* = .06). Seedlings originating from the northern region had a significantly greater maximum rhizome length (F_2,216_ = 18.39, *p* < .0001), although this was dependent on garden location. Northern plants had the longest rhizomes when grown in the mid‐latitude garden, while plants of mid‐latitude grew most poorly in the northern garden. All other treatments showed intermediate levels of rhizome growth.

### 3.3 Patterns of damage

Patterns of leaf damage confirmed that our aboveground exclosures were effective when herbivores were present. Plants exposed to aboveground interactions experienced significantly greater folivory rates at the low and mid‐latitude garden locations compared to those treated with aboveground exclosures, which had damage rates near zero (Figure S2; Tukey's grouping *p* < .05). Damage in the northern common garden also tended to be lower in exclosed treatments, but this difference was not significant (Figure S2; Tukey's grouping, *p* > .05), likely because damage at this site was low even in unexclosed plants. Plant origin did not affect rates of aboveground damage (F_2,216_ = 3.06, *p* = .051), although there were significant two and three way interactions (Table S2).

All garden locations had a small mean number of root lesions per cm of taproot, although there were some significant differences between garden locations (Figure S2; F_2,216_ = 14.48, *p* < .0001). In particular, plants grown at the northern site tended to have fewer lesions than plants at other sites (Figure S2), although exclosure treatment did not have a significant effect (i.e., the number of lesions did not differ between plants exposed to belowground interactions and those that were not; F_3,216_ = 2.51, *p* = .06). As with aboveground damage, plant origin did not affect rates of belowground damage in any garden location under any exclosure treatment (F_2,216_ = 2.70, *p *=* *.07).

### 3.4 Soil mesofaunal community

A total of 72 morphospecies were identified across all mesofaunal extraction samples: 38 Acari, 13 Collembola, 7 Insecta, and the remaining from Chilopoda, Diplopoda, Nematoda, Arachnida, and Annelida. These animals included a variety of trophic types, including predators, detritivores, and herbivores. As expected, the soil in the belowground exclosure treatments did not remain sterile over the course of the 9‐week experiment. However, the belowground exclosure treatment significantly reduced both the species richness (F_3,174_ = 24.03, *p* < .0001) and abundance (F_3,174_ = 21.72, *p* < .0001) of invertebrates in all gardens compared to the live soil treatment (Figure S3). Species accumulation curves suggest a greater sample size may have resulted in the identification of additional morphospecies (Figure S4), although meaningful comparisons can still be made between garden locations due to equal sample sizes among the three sites.

**Figure 4:**
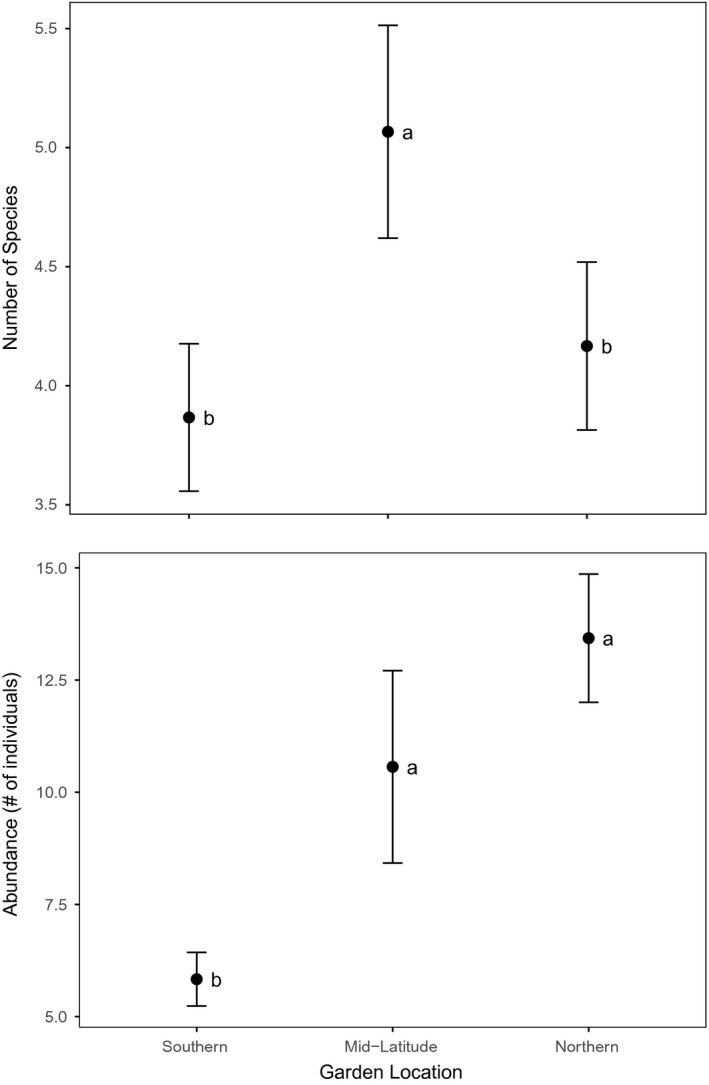
Mean ± SEM soil mesofaunal diversity (upper graph) and abundance (lower graph) extracted from 700 ml of soil using a Berlese‐Tullgren extractor (*n* = 30). Live soil treatment only

Soil mesofaunal abundance and diversity in the live soil treatments differed significantly between common garden locations (Figure 4). The southernmost garden exhibited the lowest abundance and diversity of mesofauna, differing significantly from the mid‐latitude site which exhibited the greatest abundance and diversity (Tukey's HSD, *p* < .05). Although the northern and mid‐latitude sites exhibited equally high arthropod abundances, species diversity was significantly lower at the northern site than the southern site (Tukey's HSD, *p* < .05). Similar patterns were found in soil collected from belowground exclosure treatments, although mesofaunal abundance and diversity was significantly reduced in all cases compared to live soil treatment (Figure S2).

**Figure 5:**
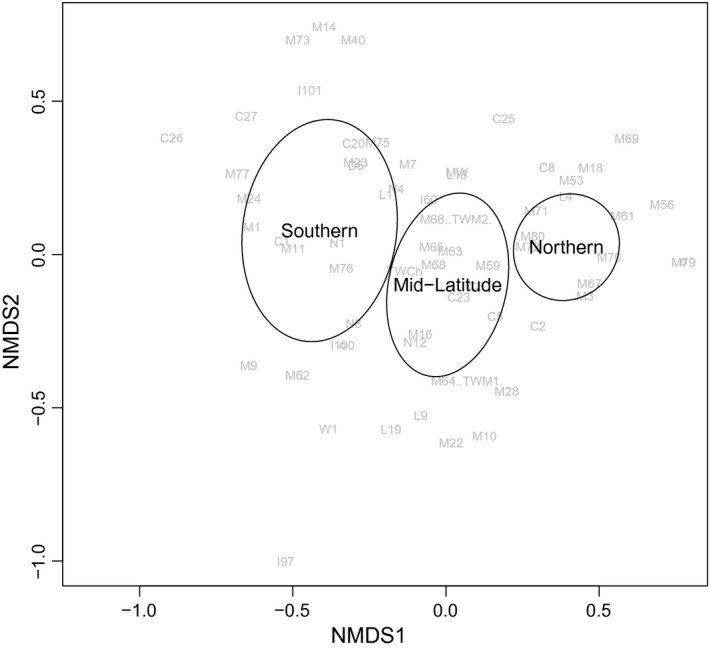
Nonmetric multidimensional scaling (NMDS) ordination plot of soil mesofaunal community following a 9‐week growing period in three common gardens. NMDS performed using Bray–Curtis percent dissimilarity of standardized abundances. Gray annotations represent unique morphospecies, and ellipses represent 95% confidence intervals. Stress value = 0.18

Among common gardens, no significant effect of plant origin was found for mesofaunal diversity (F_2,169_ = 0.71, *p* = .493) or abundance (F_2,169_ = 2.42, *p* = .092). However, a unique mesofaunal community composition was found at each common garden location (Figure 5, NMDS stress value = 0.18), with the southern and northern gardens being most dissimilar from each other. The northern garden exhibited the greatest within‐site community compositional similarity between samples. Due to low occurrences of mesofauna, NMDS analysis of sterile soil treatments was unreliable and thus not included (stress value > 0.20).

Although many of our soil fauna were not identified to the species level, we are able to comment on differences in the presence and abundance of orders and suborders of the most common taxa (Table S3). At the southern common garden location, the most diverse and abundant taxa found in the live soil treatment belonged to the orders Acari (mites) and Collembola (springtails). The most abundant Acari morphospecies was a small (~200 μm), brown, sclerotized mite of the suborder Oribatida. Oribatids can use a wide variety of food sources, including plant material, fungi material, and lichens (Siepel & De Ruiter‐Dijkman, 1993). The most common Collembola morphospecies likely belonged to the suborder Poduromorpha. Although Collembola typically do not directly consume plant material, they act as detritivores and microbivores, and thus may indirectly affect plant performance by altering the soil microbial community (Thimm, Hoffman, Borkott, Munch, & Tebbe, 1998). Collembola was the most common order found in the sterile soil treatment in the southernmost garden, with the most abundant morphospecies being the same as the live soil treatment.

At the mid‐latitude common garden location, the orders Acari and Collembola were most diverse in the live and sterile soil treatments, although Collembola were most abundant in both treatments. The most abundant Collembola morphospecies belonged to the suborder Symphypleona. The most common Acari morphospecies was a medium (~1 mm), white, soft‐bodied mite belonging to the suborder Prostigmata.

At the northern common garden location, Acari was the most abundant and diverse order in the live soil treatments, with the most abundant morphospecies being a small (200‐300 μm), brown, sclerotized mite of the suborder Oribatida. Collembola were also equally abundant as Acari in the sterile soil treatments, with the most common morphospecies being the same Symphypleona springtail as found in the mid‐latitude common garden.

## 4 DISCUSSION

With a few limited exceptions (e.g., Maron, 1998; Masters, Jones, & Rogers, 2001), ours is one of the first studies to directly compare the relative effects of above‐ and belowground interactions on an invader in a field setting, and the first to test how this relationship may change across the invaded range. Our results support the hypothesis that belowground interactions have a stronger negative impact on the performance of the invasive *C. arvense* than aboveground interactions. We also hypothesized that the aboveground–belowground relationship may change across the invaded range, potentially due to lessened herbivory in marginal populations. However, the results did not support this prediction; the relative importance of above‐ and belowground interactions on plant performance remained consistent among all three common garden locations.

### 4.1 The relative importance of above‐ and belowground interactions

As predicted, the greatest negative impact on plant growth was observed in *C. arvense* plants exposed to both above‐ and belowground enemies. However, belowground interactions generally had a greater negative impact on performance than aboveground herbivory alone. This pattern held true for all common garden locations and for all plant origins. No evidence of local adaptation to herbivory was detected, but instead the data suggest that the local environment was more important in determining herbivory rates than plant genotype. Consistent with other studies emphasizing the importance of belowground herbivory on plant performance, these findings suggest that belowground interactions alone may be as or more important in describing invader success than aboveground interactions. For instance, Barber et al. (2011) found that the performance of *Cucumis sativus* (cucumber) declined only when attacked by the root‐feeding larval form of *Acalymma vittatum* (striped cucumber beetle), and not when attacked by the leaf‐feeding adult form of the same herbivore. Although Barber et al. (2011) manipulated only a single herbivore in a controlled environment, the study provides evidence that a herbivore has the potential to impact a plant's performance more strongly by attacking belowground tissue than aboveground tissue. In our experiment, live soil treatments included the entire subterranean community, which potentially includes both mutualists and enemies. Nonetheless, a strong negative net impact of belowground interactions was still detected. Although most biological control efforts for *C. arvense* focus on the use of aboveground herbivores (Cripps et al., 2011), our results suggest that enemies targeting belowground tissue may cause a more severe decline in *C. arvense* performance.

### 4.2 Latitudinal variation in damage and invader performance

Enemy release is rarely absolute for invading plant species as over time invaders will often accumulate a new suite of enemies within their new range (Mitchell, Blumenthal, Jarosik, Puckett, & Pysek, 2010). However, the abundance and diversity of these above‐ and belowground enemies can vary greatly within and across the invader's distribution. In particular, invaders may escape enemies in marginal populations as they, for example, approach a species' northern range limit. In Ontario, herbivore damage to the non‐native common burdock (*Arctium minus*) declines rapidly with latitude (Kambo & Kotanen, 2014), and also varies locally among habitats (Lee & Kotanen, 2017). Such variation in damage can be linked with invasiveness; for example, in a greenhouse experiment, Engelkes et al. (2008) found evidence of reduced aboveground herbivory by a generalist herbivore and reduced negative soil feedback in range‐expanding plant species compared to species native to northern regions. Our results did suggest that above‐ and belowground enemies were less prominent in northern sites: Leaf damage and root lesions potentially caused by mesofaunal herbivores both were lowest in our most northern garden. However, our results did not show a change in the relative importance of aboveground–belowground interactions across a 509‐km linear distance of the *C. arvense* invaded range. Regardless of common garden location, plants consistently performed worse when exposed to belowground interactions than aboveground interactions, and worst of all when exposed to both.

### 4.3 The role of soil biota

The negative effects of soil biota are clear in our experiment, although the causal organisms are unknown. The most abundant mesofaunal morphospecies in each of the soil treatments has been identified to suborder, but without species identity, their role in the soil food web and resulting impact on *C. arvense* performance would be speculative. Although the negative effect of belowground interactions may be driven by an accumulation of herbivores or pathogens in invaded soil, the lowest abundance and diversity of soil mesofauna (including herbivores, detritivores, and predators) was observed in the southern common garden, which was also the site where the greatest reduction in above‐ and belowground biomass occurred for plants exposed to the belowground community. It still may be that particular herbivores in the soil community contribute to this negative effect, even if this is not apparent in the soil community as a whole. Southern Ontario is an agricultural region where *C. arvense* presumably has been established the longest and is the most widespread and abundant (Moore, 1975). A longer invasion history may have increased the relative occurrence of *C. arvense‐*dependent herbivores (Hawkes, 2007; Mitchell et al., 2006), or reduced the abundance and diversity of native mesofaunal species. Reduced aboveground arthropod diversity has been found in invaded plant communities (e.g., Ernst & Cappuccino, 2005; Gratton & Denno, 2006; Hagen, Bakker, & Gara, 2010), and a study by Pritekel, Whittemore‐Olson, Snow, and Moore (2006) found reduced belowground arthropod density and reduced mite diversity in plots invaded by *C. arvense* and the exotic leafy spurge (*Euphorbia esula*) compared to uninvaded plots. However, due to variation among taxa, there is no consensus on the effect of invasive plants on soil communities (Pehle & Schirmel, 2015; St John, Wall, & Hunt, 2006). Finally, the abundance of enemies found on an invasive plant may also relate to the surrounding community diversity. Bezemer et al. (2004) found more aphids and subterranean nematodes on *C. arvense* individuals when plant species diversity was high. In surveys of the surrounding community, our southern common garden was predominately surrounded by extensive *C. arvense* populations and low‐diversity *Solidago* meadows, perhaps contributing to the lower diversity of mesofauna found at this location. More knowledge of the specific *Cirsium*‐associated component of the soil community is required to elucidate its effects on performance.

Alternatively, the lack of a clear association between soil fauna and plant performance may suggest the negative responses of *C. arvense* to soil biota instead were driven by microbial pathogens not measured in this study. We chose to focus primarily on plant–animal interactions both above and below ground, but are well aware that microbial interactions can have important effects on plant performance (Dawson et al., 2016; van der Putten et al., 2001). These other components of the soil community may have contributed to the consistently negative effects of the soil biota that we observed. As with the soil fauna, microbes may both respond to and drive changes in plant populations. There is evidence of pathogen accumulation and negative soil feedback development in invasive plant populations (e.g., Diez et al., 2010; Nijjer, Rogers, & Siemann, 2007), and the strength of these interactions may be dependent on time since invasion. For instance, Lau and Suwa (2016) found performance of the invasive *Vicia villosa* was reduced when plants were inoculated with soil collected from older invasion sites compared to that from more recently colonized areas, suggesting that the soil microbial community may change over time in a way that reduces the performance of this exotic species. *C. arvense* may also undergo negative feedback from soil microbiota (Nunes, Fitzpatrick, and Kotanen, *in prep*). Thus, as exotic species become established in their invaded range, they may experience changing plant–soil feedbacks as enemies and/or mutualists accumulate over time (Mackay & Kotanen, 2008; Wolfe & Klironomos, 2005).

In summary, despite the complex biotic interactions that are inherent to this in situ experimental design, strong negative effects of both aboveground and particularly belowground communities were consistently detected across sites spanning a 500‐km latitudinal range. Our results stress the importance of including plant–soil interactions as well as aboveground interactions in studies of plant community dynamics and invader performance.

## CONFLICT OF INTEREST

The authors declare no conflicts of interest.

## AUTHOR'S CONTRIBUTIONS

All authors contributed to the development of research questions, the methodological design, and the writing of the manuscript. K.A.N. collected and analyzed the data. All authors give final approval for publication.

## DATA ACCESSIBILITY

Data available from the Dryad Digital Repository: https://doi.org/10.5061/dryad.d24pj

## Supporting information


** **
