# Supplementary material for: Comparative impacts of aboveground and belowground enemies on an invasive thistle
Source: Ecol Evol. 2017 Dec 27;8(3):1430–40. doi: 10.1002/ece3.3751 (PMC5792520; doi:10.1002/ece3.3751)
Supplement: Supplementary file 1 [file ECE3-8-1430-s001.docx]

**SUPPLEMENTARY INFORMATION**

**COMPARATIVE IMPACTS OF ABOVEGROUND AND BELOWGROUND ENEMIES ON AN INVASIVE THISTLE**

Krystal A. Nunes* and Peter M. Kotanen

Department of Ecology & Evolutionary Biology

University of Toronto Mississauga

3359 Mississauga Road

Mississauga, ON, L5L 1C6 CANADA

krystal.nunes@mail.utoronto.ca

Tel: 1-905-828-5304

Fax: 905-828-3792


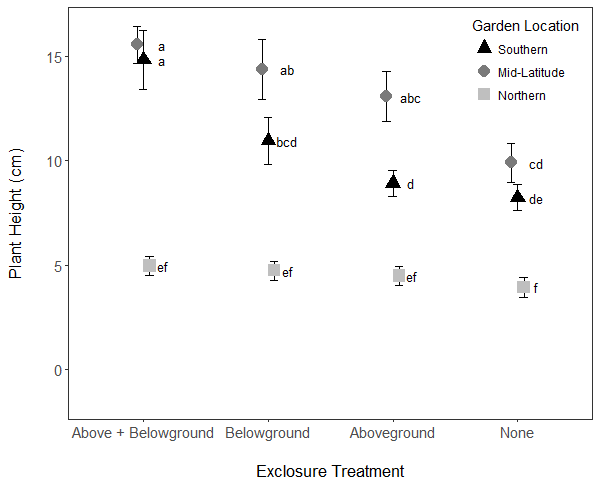


**Fig. S1.** Mean ± SEM height of *Cirsium arvense* following a 9-week growing period. Data points not sharing the same letter significantly differ from one another (Tukey post hoc, p < 0.05).


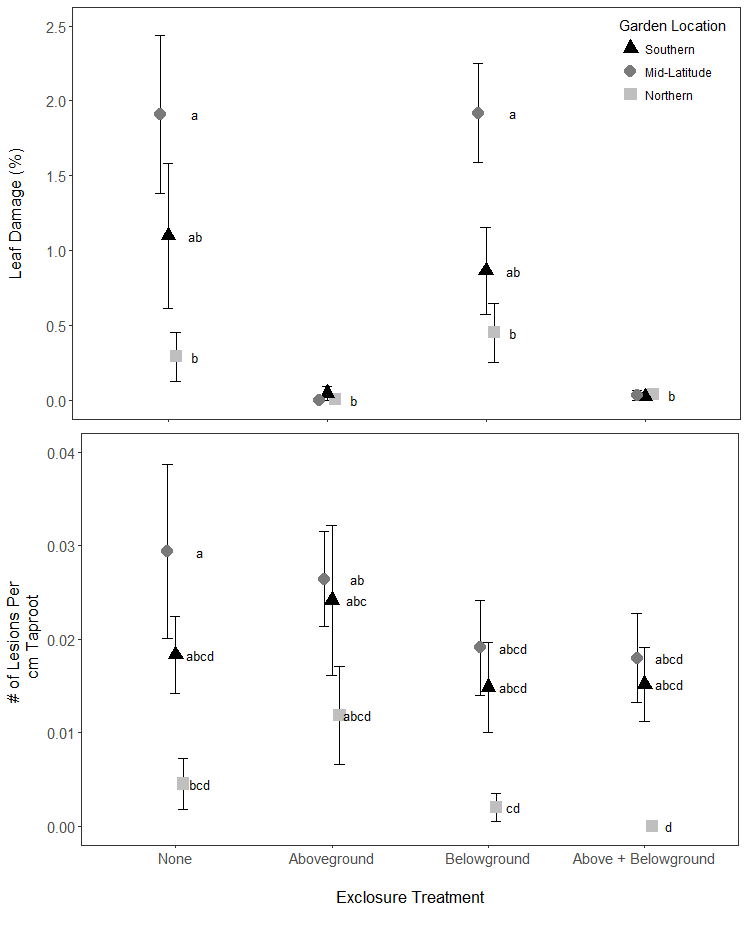


**Fig. S2.** Mean ± SEM leaf herbivory (top) and root herbivory (bottom) of *Cirsium arvense* following a 9-week growing period. Data points not sharing the same letter significantly differ from one another (Tukey post hoc, p < 0.05).

**Fig. S3.** The efficacy of the belowground exclosure treatment in reducing arthropod abundance and diversity. Mean ± SEM soil mesofaunal diversity (top graph) and abundance (bottom graph) extracted from 700 mL of soil using a Berlese-Tullgren extractor (n=30). For each category of aboveground exclosure, belowground exclosure treatments are pooled.

Extrapolated number of species

**Fig. S4.** Species accumulation curves (SAC) ± 95% CI of soil mesofaunal communities for the live soil treatments only. Analysis was performed using the function ‘specaccum’ in the vegan package in R (version 3.3.2). The "random" accumulator function was used, which finds the mean SAC and its standard deviation from random permutations of the data or subsampling without replacement (Gotelli & Colwell 2001). Curves separated by common garden location.

**Table S1.** Results of 3-way ANOVA analyses for aboveground and belowground biomass of *Cirsium arvense*.

| **Log Aboveground Biomass (g)** | **DF** | **Sum of Squares** | **F Ratio** | **Prob > F** |
| --- | --- | --- | --- | --- |
| Garden Location | 2 | 31.10 | 79.92 | <.0001 |
| Treatment | 3 | 22.71 | 38.91 | <.0001 |
| Plant Origin | 2 | 23.06 | 59.27 | <.0001 |
| Garden Location*Treatment | 6 | 2.61 | 2.24 | 0.041 |
| Garden Location*Plant Origin | 4 | 3.70 | 4.75 | 0.001 |
| Treatment*Plant Origin | 6 | 2.65 | 2.27 | 0.038 |
| Garden Location*Treatment*Plant Origin | 12 | 6.46 | 2.77 | 0.002 |
| **Log Belowground Biomass (g)** | **DF** | **Sum of Squares** | **F Ratio** | **Prob > F** |
| Garden Location | 2 | 36.72 | 106.21 | <.0001 |
| Treatment | 3 | 18.13 | 34.95 | <.0001 |
| Plant Origin | 2 | 25.91 | 74.93 | <.0001 |
| Garden Location*Treatment | 6 | 1.21 | 1.17 | 0.325 |
| Garden Location*Plant Origin | 4 | 3.16 | 4.58 | 0.001 |
| Treatment*Plant Origin | 6 | 3.81 | 3.67 | 0.002 |
| Garden Location*Treatment*Plant Origin | 12 | 2.65 | 1.28 | 0.233 |

**Table S2.** Results of 3-way ANOVA analyses for rates of aboveground and belowground herbivory of *Cirsium arvense*.

| **Leaf Damage (%)** | **DF** | **Sum of Squares** | **F Ratio** | **Prob > F** |
| --- | --- | --- | --- | --- |
| Garden Location | 2 | 25.10 | 10.45 | <.0001 |
| Treatment | 3 | 71.34 | 19.80 | <.0001 |
| Plant Origin | 2 | 7.35 | 3.06 | 0.050 |
| Garden Location*Treatment | 6 | 26.49 | 3.68 | 0.002 |
| Garden Location*Plant Origin | 4 | 23.29 | 4.85 | 0.001 |
| Treatment*Plant Origin | 6 | 7.97 | 1.11 | 0.360 |
| Garden Location*Treatment*Plant Origin | 12 | 29.23 | 2.03 | 0.023 |
| **Mean Lesions Per Cm Taproot** | **DF** | **Sum of Squares** | **F Ratio** | **Prob > F** |
| Garden Location | 2 | 0.02 | 14.48 | <.0001 |
| Treatment | 3 | 0.004 | 2.51 | 0.060 |
| Plant Origin | 2 | 0.003 | 2.70 | 0.070 |
| Garden Location*Treatment | 6 | 0.001 | 0.24 | 0.962 |
| Garden Location*Plant Origin | 4 | 0.001 | 0.42 | 0.794 |
| Treatment*Plant Origin | 6 | 0.003 | 0.96 | 0.453 |
| Garden Location*Treatment*Plant Origin | 12 | 0.01 | 1.68 | 0.072 |

**Table S3.** Species richness (top) and abundance (bottom) of soil mesofauna separated by taxa for sterile and live soil treatments in each common garden location. Results reported as mean ± SE.

| **Garden Location** | **Southern** | | **Mid-Latitude** | | **Northern** | |
| --- | --- | --- | --- | --- | --- | --- |
| *Exclosure Treatment* | *Live* | *Sterile* | *Live* | *Sterile* | *Live* | *Sterile* |
| Acari | 1.63 ± 0.24 | 0.37 ± 0.10 | 2.03 ± 0.26 | 1.5 ± 0.21 | 2.90 ± 0.29 | 0.87 ± 0.17 |
| Collembola | 1.33 ± 0.16 | 1.07 ± 0.20 | 1.80 ± 0.23 | 1.63 ± 0.19 | 1.13 ± 0.17 | 0.73 ± 0.15 |
| Insecta | 0.10 ± 0.06 | 0 | 0.07 ± 0.05 | 0.13 ± 0.08 | 0 | 0.10 ± 0.06 |
| Chilopoda | 0 | 0 | 0.10 ± 0.06 | 0.07 ± 0.05 | 0 | 0 |
| Diplopoda | 0.07 ± 0.05 | 0 | 0 | 0 | 0 | 0 |
| Nematoda | 0.40 ± 0.09 | 0.10 ± 0.06 | 0.67 ± 0.14 | 0.17 ± 0.07 | 0.10 ± 0.06 | 0.40 ± 0.10 |
| Arachnida | 0 | 0.03 ± 0.03 | 0 | 0 | 0 | 0 |
| Annelida | 0.23 ± 0.08 | 0 | 0.3 ± 0.09 | 0.03 ± 0.03 | 0 | 0.07 ± 0.05 |
| Larvae* | 0.10 ± 0.06 | 0.03 ± 0.03 | 0.1 ± 0.06 | 0.03 ± 0.03 | 0.03 ± 0.03 | 0.07 ± 0.05 |
| Total # of Species | 3.86 ± 0.31 | 1.60 ± 0.22 | 5.07 ± 0.45 | 3.57 ± 0.35 | 4.17 ± 0.35 | 2.23 ± 0.25 |
| **Garden Location** | **Southern** | | **Mid-Latitude** | | **Northern** | |
| *Exclosure Treatment* | *Live* | *Sterile* | *Live* | *Sterile* | *Live* | *Sterile* |
| Acari | 2.3 ± 0.46 | 0.37 ± 0.10 | 2.63 ± 0.41 | 1.60 ± 0.26 | 11.07 ± 1.32 | 0.93 ± 0.19 |
| Collembola | 1.97 ± 0.30 | 1.07 ± 0.20 | 5.47 ± 1.71 | 2.60 ± 0.42 | 2.23 ± 0.46 | 1.13 ± 0.22 |
| Insecta | 0.10 ± 0.06 | 0 | 0.13 ± 0.10 | 0.13 ± 0.08 | 0 | 0.10 ± 0.06 |
| Chilopoda | 0 | 0 | 0.10 ± 0.06 | 0.07 ± 0.05 | 0 | 0 |
| Diplopoda | 0.07 ± 0.05 | 0 | 0 | 0 | 0 | 0 |
| Nematoda | 0.83 ± 0.29 | 0.10 ± 0.06 | 1.13 ± 0.26 | 0.17 ± 0.07 | 0.10 ± 0.06 | 0.50 ± 0.13 |
| Arachnida | 0 | 0.03 ± 0.03 | 0 | 0 | 0 | 0 |
| Annelida | 0.47 ± 0.20 | 0 | 1.0 ± 0.49 | 0.03 ± 0.03 | 0 | 0.07 ± 0.05 |
| Larvae* | 0.10 ± 0.06 | 0.03 ± 0.03 | 0.10 ± 0.06 | 0.03 ± 0.03 | 0.03 ± 0.03 | 0.07 ± 0.05 |
| Total # of Individuals | 5.83 ± 0.60 | 2.1 ± 0.42 | 10.57 ± 2.14 | 4.63 ± 0.56 | 13.43 ± 1.43 | 2.80 ± 0.33 |

** identified larvae belonged to the orders Coleoptera and Diptera*

**REFERENCES**

Gotelli, NJ, and RK Colwell (2001) Quantifying biodiversity: procedures and pitfalls in measurement and comparison of species richness. Ecology Letters 4:379–391.
